# Efficacy of Lactobacillus and Bifidobacterium in Allergic Rhinitis: A Narrative Review

**DOI:** 10.7759/cureus.87644

**Published:** 2025-07-10

**Authors:** Tejaswi Mishra, KSBS Krishna Sasanka, Sree Sudha TY, Gulistan Bano, Swaha Panda, M Mohan Raj, Himel Mondal, Pradosh Kumar Sarangi

**Affiliations:** 1 Otorhinolaryngology-Head and Neck Surgery, All India Institute of Medical Sciences, Deoghar, Deoghar, IND; 2 Pharmacology, All India Institute of Medical Sciences, Deoghar, Deoghar, IND; 3 Physiology, All India Institute of Medical Sciences, Deoghar, Deoghar, IND; 4 Radiodiagnosis, All India Institute of Medical Sciences, Deoghar, Deoghar, IND

**Keywords:** allergic rhinitis, bifidobacterium, complementary therapy, gut-lung axis, immunomodulation, lactobacillus, microbiota, probiotics and microbiome, th1/th2 balance

## Abstract

Allergic rhinitis (AR) is a common chronic inflammatory condition of the nasal mucosa, driven by IgE-mediated immune responses to inhaled allergens. Despite the widespread use of antihistamines, steroids, and allergen immunotherapy, many patients continue to experience significant symptom burden, prompting interest in complementary therapies. Recent insights into the gut-lung axis and the immunoregulatory role of the intestinal microbiota have drawn attention to probiotics as a potential adjunct in AR management.

This narrative review synthesizes current evidence on the therapeutic utility of two extensively studied probiotic genera, *Lactobacillus* and *Bifidobacterium*, in the context of AR. Preclinical studies in murine models have demonstrated that specific strains can attenuate allergic responses by modulating cytokine profiles, restoring the Th1/Th2 balance, enhancing regulatory T cell activity, and reducing serum IgE levels. Clinical data, though heterogeneous, suggest that selected strains may improve nasal symptoms and quality of life in both adult and pediatric populations. Meta-analyses support these findings, but they also highlight considerable variation in strain specificity, dosage, treatment duration, and patient characteristics across studies. The overall safety profile of these probiotics remains favorable, with minimal adverse effects reported.

Although evidence supports a potential role for *Lactobacillus* and *Bifidobacterium* in AR management, inconsistencies and methodological limitations underline the need for more robust, strain-specific randomized controlled trials. Future research should focus on developing standardized protocols, defining clinical endpoints, and conducting mechanistic studies to elucidate their role in immunomodulation.

## Introduction and background

Allergic rhinitis (AR), a frequent inflammatory condition affecting the nasal mucosa, impacts a considerable fraction of the global population, with an estimated prevalence of 10% to 40% [1]. This condition is a type 1 hypersensitivity reaction to airborne allergens, manifesting as symptoms such as sneezing, rhinorrhea, nasal congestion, and itching, often accompanied by ocular symptoms. The effects of AR extend beyond physical discomfort. It often disrupts sleep quality, lowers work and academic performance, and significantly decreases overall quality of life [1].

Currently, the management of AR includes lifestyle modifications, such as avoiding allergen exposure, medications (including antihistamines and corticosteroids), and allergen-specific immunotherapy (AIT) [2]. Although medical management is effective in controlling allergic symptoms, it can cause side effects related to the aforementioned medications, and some patients may not achieve the desired results, leading to a search for complementary and alternative treatment approaches that can be used in conjunction with available medications and are safe for patients.

Among the many areas being explored, the "gut-lung axis" is one of the key options. Research has suggested that gut microbiota can influence our immune system and can have a role in the development of allergic diseases. One such hypothesis is the "microflora hypothesis," which suggests that changes in gut microbiota, also called "dysbiosis," can lead to various immune disorders, including the development of allergies [3]. Additionally, it suggests that the gut and lungs are affected due to these immune disturbances, and thus, a change in gut microbiota can influence immune responses in the lungs and nasal passages [4].

These studies paved the way for the use of probiotics to positively modulate gut microbiota and alleviate AR symptoms. Among the various probiotics tested, *Lactobacillus* and *Bifidobacterium* species have garnered considerable interest, as these species are well recognized for their ability to interact with the host immune system and influence immune responses [5]. In view of the widespread occurrence of AR and the significant economic burden, this narrative review aims to provide a comprehensive evaluation of the evidence regarding the efficacy of these microbes in treating AR.

## Review

Materials and methods

Based on the available literature and existing knowledge about the role of probiotics, particularly various strains of *Lactobacillus* and *Bifidobacterium*, in the management of AR, this narrative review aims to provide a broad and balanced overview. A thorough literature search was done, and various preclinical animal studies, individual clinical trials, systematic reviews, and meta-analyses were reviewed.

The selection criteria included studies that explored the impact of these specific probiotic strains on AR. Both human and animal studies deemed relevant were included if they provided insights into the therapeutic potential or biological activity of these probiotics in relation to AR. A structured data extraction process was used to maintain consistency across the included studies. Key variables extracted included participant characteristics, such as pediatric or adult populations, specific strains of* Lactobacillus* and/or *Bifidobacterium* administered, dosage and delivery methods, duration of intervention, and primary clinical or immunological outcomes assessed. Additionally, the main conclusions drawn by each study were also recorded to support the thematic synthesis.

The identified studies were then summarized qualitatively, rather than performing a quantitative analysis. This method enabled the identification of recurring patterns, emerging themes, and differences in study outcomes, which were critically reviewed and discussed in the following sections. By combining data from various types of evidence, this review aims to provide a comprehensive understanding of the current evidence landscape and suggest potential directions for future research in probiotic-based treatments for AR.

Discussion

AR is an immunoglobulin E (IgE)-mediated inflammatory response localized to the nasal passages, resulting from exposure to inhaled allergens. Once exposed to an allergen, sensitized mast cells in the nasal mucosa, which are coated with allergen-specific IgE, release a cascade of proinflammatory substances, such as histamine, leading to the typical immediate symptoms of AR [6]. Following this early phase, a late-phase response begins, leading to the infiltration of various inflammatory cells, including eosinophils, T-lymphocytes, and basophils, into the nasal mucosa, contributing to the ongoing inflammation and congestion in the nasal mucosa [7]. Thus, these complex immune mechanisms offer various possible targets for therapeutic intervention.

Additionally, an imbalance between the T helper type 1 (Th1) and T helper type 2 (Th2) immune responses is another key aspect of the pathophysiology of AR, characterized by a shift toward Th2 responses. This Th2-skewed response leads to the production of various inflammatory cytokines, such as interleukin-4 (IL-4) and interleukin-13 (IL-13), which are essential for IgE production by B cells [6,8]. In contrast to a Th2-skewed response, a balanced or Th1-dominant response usually helps suppress allergic inflammation. Thus, treatment strategies based on restoring the Th1/Th2 balance or reducing IgE production show promise for managing AR.

With increasing evidence from research, the "gut-lung axis" has provided an important and compelling link between the gastrointestinal and respiratory systems [9]. This concept emphasizes the two-way relationship between gut bacteria and the lungs, suggesting that the makeup and activity of the gut microbiota can also influence immune responses in distant organs, such as the airways and nasal cavity. Additionally, growing evidence suggests that changes in gut bacteria, also known as dysbiosis, have been linked to the initiation and exacerbation of allergic diseases, including AR [3]. Thus, this connection provides a possible biological rationale for exploring the use of orally administered probiotics, which can influence intestinal microbial composition and potentially impact the course of AR.

Overview of Lactobacillus and Bifidobacterium

Among the various studied probiotic agents, *Lactobacillus* and *Bifidobacterium* represent two major gram-positive bacterial genera that are well known for their probiotic qualities, inhabiting the human gastrointestinal tract as common commensals [3]. These bacteria employ numerous distinct mechanisms to achieve their beneficial effects, which help to regulate the host's immune system, improve the intestinal barrier's function, and produce antimicrobial substances [5]. They also interact with immune cells in the gut and influence the production of various cytokines involved in the pathophysiology of AR. This interaction functions as a probable mechanism to reestablish equilibrium between Th1 and Th2 immune responses. Additionally, it is essential to recognize that the specific effects of these probiotics on the immune system can vary significantly among strains within these genera, underscoring the need for targeted research to identify strains that are effective for specific conditions, such as AR [10].

Evidence From Preclinical and Clinical Research

Preclinical studies: For the identification of the potential role of *Lactobacillus* and *Bifidobacterium* strains in the treatment of AR, several preclinical studies using animal models of AR have been conducted. One such notable study examined the effects of *Bifidobacterium longum* IM55 and *Lactobacillus plantarum* IM76 in mice with AR caused by exposure to house dust mite allergens [8]. The findings suggested that giving these strains orally, either alone or in combination, significantly reduced allergic nasal symptoms. Their administration also resulted in lowered levels of important Th2 cytokines, including IL-4 and IL-5, in the nasal mucosa, bronchoalveolar lavage fluid (BALF), and blood. Additionally, it also lowered the levels of IgE in the blood. It was also noted that there was a reduction in the infiltration of eosinophils and mast cells into the BALF, while the number of regulatory T cells (Tregs) and the levels of the anti-inflammatory cytokine IL-10 were increased. The probiotic treatments also had a positive effect on the gut microbiota composition in these mice. These results also suggested that the specific *Lactobacillus* and *Bifidobacterium* strains can indeed alleviate AR symptoms in animal models through various immunomodulatory pathways.

Another preclinical study investigating *Bifidobacterium breve* in a murine AR model showed that giving live *B. breve* orally in doses of 107 colony-forming units (CFU) or more effectively reduced nasal mucosal injury and decreased sneezing with repeated use [11]. Moreover, when the dosages were increased, *B. breve* decreased the levels of serum OVA-specific IgE, IL-4, and IL-10 and increased the splenic proportion of CD4+CD25+ Tregs in rhinitic mice. The merits of this study included identifying the potential role of *Bifidobacterium* in managing AR and also in suggesting a possible dose-response relationship, which can be crucial for determining optimal therapeutic dosages in future clinical trials. Furthermore, *Lactobacillus paracasei*-33 has also been shown to attenuate house dust mite-induced AR in mice, further expanding the evidence for the anti-allergic potential of different strains within the *Lactobacillus* genus in preclinical settings [8].

Clinical trials: Unlike preclinical studies, the evidence base from clinical trials investigating the efficacy of *Lactobacillus* and *Bifidobacterium* in AR is more varied. A meta-analysis, synthesizing data from 28 studies, assessed the general effects of probiotics in AR, showing that they were capable of contributing to the relief of symptoms and improving the quality of life of these patients [3]. Nevertheless, the authors found considerable heterogeneity in some of the outcomes, indicating that the effects of probiotics may vary depending on several factors. This variation may be due to differences in the strain of probiotic used, the dose and duration of treatment, the nature of the patient population treated, and the types of outcome measures evaluated in the various trials.

Another meta-analysis, which evaluated pediatric AR in 28 studies involving 4,765 patients, showed that probiotic administration correlated with notable improvements in total nose symptom scores, itchy nose scores, sneezing scores, eye symptoms, and scores on the Pediatric Rhinoconjunctivitis Quality of Life Questionnaire (PRQLQ) [12]. However, this analysis was unable to identify any supportive evidence that probiotics could halt the onset of AR in the pediatric population. This distinction between the therapeutic effects on existing symptoms and the lack of a preventive effect is important for understanding the potential utility of probiotics in treating AR among children.

From extracting data from a subgroup analysis of one meta-analysis, *Lactobacillus* strains were found effective in relieving symptoms of seasonal and perennial AR [3]. Among others, one strain in particular, *Lactobacillus paracasei *LP-33, was noted to significantly contribute to the improved quality of life of patients with AR. Similarly, a review of another subgroup showed that mixed *Bifidobacterium* strains led to significantly greater relief of AR symptoms compared to a single *Bifidobacterium* strain, which was effective in improving quality of life. These findings suggest that specific strains and formulations within both genera may hold promise for alleviating AR symptoms and enhancing patient well-being.

Other individual clinical trials have also demonstrated mixed outcomes. Benefits of certain* Lactobacillus *strains (e.g.,* L. paracasei, L. acidophilus, L. johnsonii, L. salivarius*, *L. plantarum, and* *L. gasseri*) and *Bifidobacterium* species (e.g., *B. longum*, *L. bifidum*, *B. breve*, *B. lactis*, *B. infantis*, and *B. subtilis*) have been studied on the various aspects of AR, including symptom reduction and quality of life [3]. For example, *Lactobacillus paracasei*-33 fortified fermented milk intake led to improved quality of life in individuals suffering from perennial AR [13]. Another notable study demonstrated that the combination of *Lactobacillus johnsonii* EM1 with levocetirizine had superior efficacy in treating perennial AR in pediatric patients compared to using levocetirizine as a monotherapy [14]. Conversely, other clinical trials have failed to demonstrate significant benefits of probiotic treatment in AR. For example, *Lactobacillus rhamnosus* therapy was unable to yield any beneficial effect on birch pollen allergy symptoms [15]. This inconsistency in the therapeutic advantages of probiotics underscores the need for further research to identify which specific strains, at what dosage, and in which patient populations are most likely to be effective.

In a recent study done by Lungaro et al., attempting evaluation of the effect of a probiotic mixture containing *L. acidophilus*, *L. rhamnosus*, *B. breve*, and *B. longum* in adult patients with AR showed a marked improvement in MiniRQLQ (Mini Rhinitis Quality of Life Questionnaire) scores in the probiotic group compared to the placebo group [2], thus indicating an enhancement in symptom management and overall well-being. The study also observed changes in the fecal microbiota makeup in the probiotic group, with an increase in bacteria associated with anti-inflammatory properties [2]. This suggests that multi-strain probiotic formulations may be beneficial in AR and that their effects could be mediated, at least in part, through alterations in the gut microbiota.

To provide a clearer overview of the clinical evidence, we have summarized the key findings from selected clinical trials mentioned in the provided research material in Table 1.

**Table 1:** Summary of Key Clinical Trials Evaluating Lactobacillus and Bifidobacterium in Allergic Rhinitis TSS: Total symptom score; PRQLQ: Pediatric Rhinoconjunctivitis Quality of Life Questionnaire; ECP: Eosinophil cationic protein; IL: Interleukin; TNSS: Total Nasal Symptom Score Questionnaire; QoL-KCAR: Questionnaire in Korean Children with AR; NSDS: nasal symptom duration score; MiniRQLQ: Mini Rhinitis Quality of Life Questionnaire

Study	Publication Year	Probiotic Strain(s)	Dosage Form	Participants	Type of AR	Duration	Primary Outcome	Key Findings
Wang et al., [13]	2004	*Lactobacillus paracasei*-33	Fortified fermented milk	Adults	Perennial	30 days	Quality of life	Improved quality of life
Peng et al., [16]	2005	*Lactobacillus paracasei* LP-33 (live and heat-killed)	Not specified	Pediatric	Perennial	Not specified	Quality of life	Improved overall quality of life with both live and heat-killed strains
Ishida et al., [17]	2005	*Lactobacillus acidophilus* L-92	Acidified milk	Adults	Perennial	Not specified	Allergic symptoms	Alleviated symptoms
Lue et al., [14]	2012	*Lactobacillus johnsonii* EM1	Not specified	Pediatric	Perennial	3 months	TSS and PRQLQ	Decreased TSS when added to levocetirizine
Lin et al., [18]	2013	*Lactobacillus salivarius* PM-A0006	Not specified	Pediatric	Perennial	Not specified	Rhinitis symptoms and drug usage	Reduced symptoms and drug usage
Costa et al., [19]	2014	*Lactobacillus paracasei* subsp. (paracasei LP-33)	Not specified	Adults	Perennial	8 weeks	Quality of life	Improved quality of life, particularly ocular symptoms
Xiao et al., [20]	2006	*Bifidobacterium longum* BB536	Not specified	Adults	Japanese cedar pollinosis	Pollen season	Clinical symptoms and plasma cytokine levels	Relieved clinical symptoms and modulated cytokine levels
Miraglia Del Giudice et al., [21]	2017	*Bifidobacterium longum* BB536, *B. infantis* M-63, *B. breve* M-16V	Not specified	Pediatric	Seasonal	2 months	AR symptoms and quality of life	Significantly improved symptoms and quality of life
Dennis-Wall [22]	2017	*Lactobacillus gasseri* KS-13, *Bifidobacterium bifidum* G9-1, *B. longum* MM-2	Not specified	Adults	Seasonal	8 weeks	Rhinoconjunctivitis-specific quality of life	Improved quality of life
Anania et al., [23]	2021	*Bifidobacterium animalis* subsp. lactis BB12 and *Enterococcus faecium* L3	Not specified	Pediatric	Not specified	3 months	AR signs and symptoms	Decreased signs and symptoms
Helin et al., [15]	2002	*Lactobacillus rhamnosus* GG	Not specified	Adults	Birch pollen allergy	Pollen season	Allergy symptoms, medication use, and oral apple challenge	No beneficial effect
Tamura et al., [24]	2007	*Lactobacillus casei* strain *Shirota* (LcS)	Fermented milk	Adults	Japanese cedar pollen sensitivity	Pollen season	Allergic symptoms	Did not prevent allergic symptoms
Ouwehand et al., [25]	2009	*Lactobacillus acidophilus* and *Bifidobacterium lactis*	Not specified	Pediatric	Birch pollen allergy	Pollen season	Nasal symptoms and eosinophil infiltration	Prevented eosinophil infiltration and reduced nasal symptoms
Yonekura et al., [26]	2009	*Lactobacillus paracasei* strain KW3110	Not specified	Adults	Japanese cedar pollinosis	Pollen season	Nasal symptoms, ECP level, quality of life	Reduced nasal symptoms and ECP, improved quality of life when pollen count was low
Nagata et al., [27]	2010	*Lactobacillus plantarum* No. 14 (LP14)	Not specified	Adults	Seasonal	Not specified	Gene expression of Th1-type cytokines	Induced gene expression of Th1 cytokines
Nembrini et al., [28]	2015	*Lactobacillus paracasei* NCC 2461	Not specified	Adults	AR	8 weeks	Nasal symptoms	No beneficial effect
Kang et al., [29]	2020	*Bifidobacterium longum* and *Lactobacillus plantarum* (NVP-1703)	Not specified	Adults	Perennial	8 weeks	AR symptoms and IL-10 expression	Relieved symptoms by inducing IL-10
Singh et al., [30]	2013	*Bifidobacterium lactis* NCC2818	*B. lactis* NCC2818 4 × 10^9^ CFU per day blended in maltodextrin	Adults	Seasonal	8 weeks	TNSS Score	TNSS scores decreased significantly at 8 weeks of treatment
Jeong et al., [31]	2024	*B. longum* IM55 and *L. plantarum* IM76	Not specified	Pediatric and adolescents (6–19 years)	Perennial	4 weeks	TNSS, NSDS, and QoL-KCAR Scores	TNSS, NSDS, and QoL-KCAR scores decreased significantly
Lungaro et al., [2]	2024	*Lactobacillus acidophilus* PBS066, *L. rhamnosus* LRH020, *B. breve* BB077, *B. longum* subsp. *longum* BLG240	Probiotic food supplement containing *L. acidophilus* PBS066 1 × 10^9^ UFC, *L. rhamnosus* LRH020 1 × 10^9^ UFC, *B. breve* BB077 1 × 10^9^ UFC, and *B. longum* BLG240 1 × 10^9^ UFC plus excipients (maltodextrin, corn starch, and magnesium salt of fatty acids)	Adults	Persistent or seasonal	8 weeks	MiniRQLQ score	Significant improvement in MiniRQLQ score

Impact on the Th1/Th2 Balance and IgE Production in AR

A potential mechanism through which probiotics might exert their effects in AR is by modulating the equilibrium between Th1 and Th2 immune responses. Given that allergic conditions are often associated with a Th2-dominant immune profile, interventions that can shift this balance toward Th1 may be beneficial. Indeed, some evidence suggests that probiotics can increase the Th1/Th2 ratio in patients with AR. Several systematic reviews and meta-analyses have demonstrated an increase in this ratio within the probiotic treatment group compared to the placebo group [3,32,33].

However, not all studies have shown a consistent impact of probiotics on IgE levels, a key antibody involved in allergic reactions. Several meta-analyses and individual studies have reported no significant changes in overall or antigen-specific IgE levels following probiotic treatment. This suggests that the potential benefits of probiotics in AR may not rely solely on the reduction of IgE production but could involve other immunomodulatory mechanisms [32-34]. These alternative possible mechanisms could include influencing the production of various cytokines or modulating the activity of different immune cell populations, such as regulatory T cells. A recent network analysis evaluating the effectiveness of various probiotics in decreasing total IgE levels indicated that *Bifidobacterium*, followed by *Lactobacillus* and *Tetragenococcus halophilus*, outperformed other probiotic strains and other conventional treatments [35]. Furthermore, the study revealed that a *Bifidobacterium* mixture (*B. longum* BB536, *B. breve* M-16V, and *B. infantis* M-63) exhibited the greatest efficacy in reducing RQLQ scores.

Based on the evidence found in preclinical studies, *Lactobacillus* and *Bifidobacterium* strains have been found to exert specific effects on cytokine production. Based on a murine model of AR, *B. longum* and *L. plantarum* reduced the production of Th2-associated cytokines, namely IL-4 and IL-5, but on the other hand, increased the production of IL-10, an anti-inflammatory regulatory cytokine [8]. This underscores their potential to alleviate symptoms of AR.

Safety and Delivery Modalities

The safety record of *Lactobacillus* and *Bifidobacterium* supplementation seems to be quite positive, with most clinical studies showing no serious side effects associated with these probiotics in people with AR. Even when adverse events are reported, they are usually mild and transient, often involving gastrointestinal symptoms such as diarrhea, abdominal pain, or flatulence [36]. This favorable safety profile thus presents an important advantage for considering probiotics as a potential treatment option for AR.

*Lactobacillus* and *Bifidobacterium* strains are typically administered in oral forms, which can take various forms, including capsules, tablets, or as components of fermented foods [2]. When considering a mix of these strains, as in a clinical study examining a combination of *L. acidophilus*, *L. rhamnosus*, *B. breve*, and *B. longum*, the probiotics were administered in capsule form. Participants were instructed to take one capsule daily [2]. This ease of oral administration makes these probiotics a convenient option for potential therapeutic use in managing AR.

Challenges in Clinical Implementation and Future Directions

Several challenges exist in using probiotics for AR in practice, which warrant mention despite the promising findings from some preclinical and clinical studies. Among these, one of the major obstacles is the significant differences observed in various studies, including study design, specific probiotic strains and dosages, treatment duration, and outcome measures evaluated. This variability makes it challenging to draw stable and consistent conclusions about the effectiveness of probiotics for AR and to provide clear clinical recommendations.

Furthermore, the evidence supporting important outcomes, such as symptoms and quality of life, is considered low to very low in several systematic reviews and meta-analyses. This highlights the need for more thorough and high-quality research to establish the evidence base and determine the real clinical benefits of probiotics in AR.

Future research in this area should focus on well-designed, randomized controlled trials with larger sample sizes, standardized dosages and treatment lengths, and consistent, clinically relevant outcome measures. It would be beneficial to focus on specific probiotic strains or combinations that have demonstrated promise in preclinical studies or early clinical trials. Additionally, more investigation into how these probiotics work in the context of AR is necessary. This includes studying their effects on gut microbiota, specific immune cell groups, and cytokine profiles in patients with AR.

Subgroup analyses are another aspect in which future studies should aim to determine if specific patient groups, based on factors such as age, the type of AR (seasonal vs. perennial), or the presence of other conditions, are more likely to benefit from probiotic supplementation. This could help in customizing treatment recommendations and moving toward a more personalized approach to managing AR.

## Conclusions

The current body of evidence suggests that supplementation with *Lactobacillus *and *Bifidobacterium* strains may help relieve AR symptoms and improve the quality of life for those affected. Meta-analyses and several clinical trials have shown positive results, especially with specific strains and formulations. However, the findings are not completely consistent, and significant heterogeneity across studies necessitates a careful interpretation of the current evidence. The generally good safety profile of these probiotics makes them an appealing option for further research. Well-designed clinical trials are crucial for drawing more definitive conclusions and informing the potential use of *Lactobacillus* and *Bifidobacterium* as additional or alternative treatments for managing AR.
